# AIMS65 scoring system is comparable to Glasgow-Blatchford score or Rockall score for prediction of clinical outcomes for non-variceal upper gastrointestinal bleeding

**DOI:** 10.1186/s12876-019-1051-8

**Published:** 2019-07-26

**Authors:** Min Seong Kim, Jeongmin Choi, Won Chang Shin

**Affiliations:** 1 Department of Internal Medicine and Liver Research Institute, Seoul National University College of Medicine, 101, Daehak-Ro, Jongno-gu, Seoul, 03080 South Korea; 2 Department of Internal Medicine, Sanggye Paik Hospital, Inje University College of Medicine, 1342 Dongil-ro, Nowon-gu, Seoul, 01757 South Korea

**Keywords:** Stomach, Gastrointestinal hemorrhage, AIMS65 score, Glasgow-Blatchford score, Rockall score

## Abstract

**Background:**

Risk stratification for patients with nonvariceal upper gastrointestinal (NVUGI) bleeding is crucial for successful prognosis and treatment. Recently, the AIMS65 score has been used to predict mortality risk and rebleeding. The purpose of this study was to compare the performance of the AIMS65 score with the Glasgow-Blatchford score (GBS), Rockall score, and pre-endoscopic Rockall score in Korea.

**Methods:**

We retrospectively studied 512 patients with NVUGI bleeding who were treated at a university hospital between 2013 and 2016. The AIMS65, GBS, Rockall score, and pre-endoscopic Rockall score were used to stratify patients based on their bleeding risk. The primary outcome was in-hospital mortality. The secondary outcomes were composite clinical outcomes of mortality, rebleeding, and intensive care unit (ICU) admission. Each scoring system was compared using the receiver-operating curve (ROC).

**Results:**

A total of 17 patients (3.3%) died and rebleeding developed in 65 patients (12.7%). Eighty-six patients (16.8%) required ICU admission. The AIMS65 (area under the curve (AUC) 0.84, 95% confidence interval, 0.81–0.88)) seemed to be superior to the GBS (AUC 0.72, 0.68–0.76), the Rockall score (AUC 0.75, 0.71–0.79), or the pre-endoscopic Rockall score (AUC 0.74, 0.70–0.78) in predicting in-hospital mortality, but there was not a statistically significant difference between the groups (*P* = 0.07). The AUC value of the AIMS65 was not significantly different from the other scoring systems in prediction of rebleeding, endoscopic intervention, or ICU admission.

**Conclusions:**

The AIMS65 score in NVUGI bleeding patients was comparable to the GBS or Rockall scoring systems when predicting the mortality, rebleeding, or ICU admission. Because AIMS65 is a much easier, readily calculated scoring system compared to the others, we would recommend using the AIMS65 in daily practice.

## Background

Upper gastrointestinal (UGI) bleeding is a medical emergency with an incidence of mortality of 5–10% [1]. The guidelines recommend use of risk stratification tools in UGI bleeding to facilitate accurate triage and assist in clinical decisions such as endoscopic timing and level of care [2, 3]. It is important for physicians to identify UGI bleeding patients who are at high risk of mortality or rebleeding.

There are some scoring systems have been developed to predict bleeding outcomes for patients with UGI bleeding. The Rockall score (RS) and the Glasgow-Blatchford risk score (GBS) are the most widely-used scoring systems in clinical practice [4, 5]. These scoring systems have been reported to be useful in predicting mortality, rebleeding, need for transfusion, and hemostasis [6, 7]. However, there are limitations in these scoring systems. The GBS is difficult to calculate in routine clinical practice due to complex nature of score calculation, and the RS requires endoscopic findings.

Recently, much interest has been shown in pre-endoscopic risk scores for UGI bleeding. The pre-endoscopy Rockall score (PRS) excludes the endoscopic findings that are needed for the RS; because of this, using the PRS to make clinical predictions, has been controversial [8, 9]. The AIMS65 scoring system was developed to determine the prognosis of patients with UGI bleeding [10]. The AIMS65 score is based on albumin levels, prothrombin time (PT), international normalized ratio (INR), altered mental status, systolic blood pressure, and whether age is 65 years and older. One point is assigned to each variable that increases the risk of clinical outcomes. Compared to other scoring systems, the AIMS65 has the advantage of being simple to perform in an emergency situation [11–13]. But AIMS65 score has not been sufficiently validated in Korea. Few studies have compared AIMS65 score with other scoring systems [11, 14]. One study suggested that AIMS65 score was not suitable for predicting the need for endoscopic intervention [14].

The causes of UGI bleeding differ among countries. The prevalence of variceal bleeding is higher in Korea than in Western countries [14]. Limited data are available on validation of scoring systems in patients with non-variceal UGI (NVUGI) bleeding in Korea.

The aim of this study was to compare the predictive value of the AIMS65 with the GBS, PRS, and RS scores for a large scale of NVUGI bleeding patients in Korea.

## Methods

### Inclusion and exclusion criteria of patients

From January 2013 to June 2016, patients for the UGI bleeding who visited the emergency medical center of the Sanggye Paik Hospital, Seoul, South Korea were reviewed retrospectively in this study. UGI bleeding was confirmed by the hospital staff based on the presence of hematemesis or melena. All patients underwent emergency upper endoscopy within 24 h. Patients with esophageal or gastric variceal bleeding were excluded. Cases of iatrogenic post-procedural bleedings after endoscopic resection for gastric tumors were also excluded. The data of the patients were collected in the electronic medical record. Patients were excluded if the data required for calculation of relevant risk stratification scores were unavailable. The data were reviewed by two endoscopy specialists (M.S.K. and J.C.) for all cases.

### Treatment of UGI bleeding

Patients received the same treatment according to emergency bleeding protocols. All patients received intravenous proton pump inhibitor infusion before upper endoscopy. Upper endoscopy was performed by endoscopy specialists within the first 24 h. Endoscopic therapies were applied as following conditions: (1) peptic ulcers with actively bleeding (type Ia), oozing hemorrhage (type Ib), or a nonbleeding visible vessel (type IIa); (2) Dieulafoy’s lesion (3) angiodyplasia (4) and any lesions with active bleeding. Adherent blood clot (type IIb), flat spot (type IIc), clean base ulcer (type III), or lesions with no active bleeding stigmata including acute gastric mucosal lesion or Mallory-Weiss tear were initially treated medication without endoscopic therapy.

Endoscopic therapies included injection of epinephrine, hemostatic forcep electrocoagulation, argon plasma coagulation, or application of endoscopic clips. In cases of failed endoscopic hemostasis, transarterial embolization was preferred over surgery. When rebleeding occurred after successful endoscopic therapy, endoscopic therapy was initially preferred. Surgery as salvage therapy was performed when embolization was unsuccessful.

Transfusion was required if hemoglobin was below 8 g/dL. ICU admission was considered in the following conditions: if the patients have any symptoms of confusion or altered mentality; presence of hemodynamic instability (systolic blood pressure < 90 mmHg, need for vasoactive drugs); or severe comorbid illness including heart failure, chronic renal failure, liver cirrhosis, or chronic lung disease. Decision of ICU admission was made finally by ICU unit attending physicians.

### Study design

The following data were collected through electronic medical record review: age, sex, symptoms of visiting emergency centers (hematemesis, melena, shock, syncope, or altered mentality), vital sign (heart rate, systolic blood pressure), the Glasgow Coma Scale, mental status, medications that contribute to bleeding (aspirin, clopidogrel, warfarin, nonsteroidal anti-inflammatory drugs, other antithrombotic agents). Laboratory findings (hemoglobin, albumin level, blood urea nitrogen, PT, and INR), and endoscopic findings were also collected. The AIMS65 score, RS, PRS, and GBS were obtained based on the data. Components of the each scoring system were described in Table 1. Primary outcome was in-hospital mortality and secondary outcomes were composite clinical outcomes of in-hospital mortality, intensive care unit (ICU) admission; rebleeding; blood transfusion requirement; and endoscopic, radiologic, or surgical intervention.

**Table 1:** Components of the AIMS65, full and pre-endoscopic Rockall, and Glasgow-Blatchford scoring system

AIMS65 score	Score	Rockall score	Score
Albumin < 3.0 mg/dL	1	Age	
INR > 1.5	1	< 60 yrs	0
Altered mental status, GCS < 14	1	60–79 yrs	1
Systolic BP < 90 mmHg	1	≥ 80 yrs	2
Age > 65 yrs	1	Shock	
Maximum score	5	No shock	0
Pre-endoscopic Rockall Score		Pulse > 100 bpm, Systolic BP > 100 mmHg	1
Age		Systolic BP < 100 mmHg	2
< 60 yrs	0	Comorbidity	
60–79 yrs	1	No major comorbidity	0
> 80 yrs	2	CHF, IHD, or major comorbidity	2
Shock		Renal failure, liver failure, metastatic cancer	3
No shock	0	Diagnosis	
Pulse > 100, Systolic BP > 100 mmHg	1	Mallory-Weiss tear or no stigmata/lesion	0
Systolic BP < 100 mmHg	2	All other diagnoses	1
Comorbidity		GI malignancy	2
No major	0	Evidence of bleeding	
CHF, IHD, or major comorbidity	2	No stigmata or dark spot on ulcer	0
Renal failure, liver failure, metastatic cancer	3	Blood in UGI tract, adherent clot, visible/spurting vessel	2
Maximum score	7	Maximum score	11
Glasgow-Blatchford score			
Blood urea, mmol/L		Systolic BP, mm Hg	
6.5–8	2	100–109	1
8–10	3	90–99	2
10–25	4	< 90	3
> 25	6	Other risk factors	
Hemoglobin, g/dL, Men		Pulse (≥100/bpm)	1
12- < 13	1	Melena	1
10- < 12	3	Syncope	1
< 10	6	Liver disease	2
Hemoglobin, g/dL, Women		Heart failure	2
10- < 12	1	Maximum score	23
< 10	6		

*INR* International normalized ratio, *GCS* Glasgow Coma Scale, *BP* Blood pressure, *CHF* Congestive heart failure, *IHD* Ischemic heart disease, *bpm* beat per minute, *UGI* Upper gastrointestinal

### Statistical analysis

The performance of each scoring system was evaluated with a receiver operating characteristic (ROC) curve, calculation of area under the curve (AUC) with 95% confidence intervals. In a ROC curve the true positive rate (sensitivity) is plotted in function of the false positive rate (100-specificity) for different cut-off points of a parameter. Each point on the ROC curve represents a sensitivity/specificity pair corresponding to a particular decision threshold. The cut-off value of each scoring system is determined by the ROC curve with the most specificity and sensitivity. The risk prediction scoring system was divided into two groups, high-risk group and low-risk group. For each scoring systems, a cutoff point was calculated that maximized the sum of sensitivity and specificity in predicting the primary and secondary outcomes. Fischer’s exact test was used for categorical variables. All confidence intervals are described as two-sided binomial 95% confidence intervals. The cut-off value 0.05 considered to be the threshold for statistical significance. In a Bonferroni correction to adjust for the multiple comparisons in the scoring systems (in which the *P* values were divided by 4 for the comparison with an alpha level of 0.05), the corrected *P* values were 0.0125 for the combination group versus. Data were analyzed using SPSS Statistics for Windows (version 23.0; IBM Corp., Armonk, NY, USA). AUCs were compared with the DeLong method by using MedCalc program (version 16.8.4; MedCalc Software, Mariakerke, Belgium).

## Results

### Baseline characteristics

**Fig. 1:**
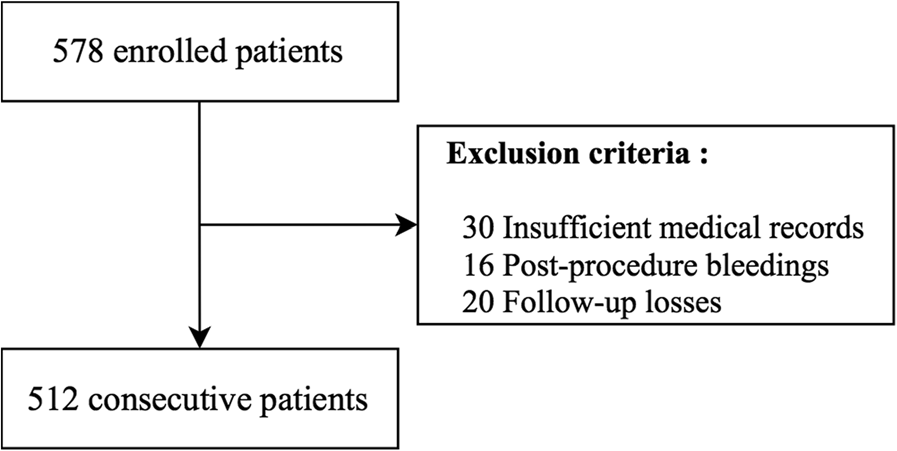
Study flow-chart

A total of 578 patients were enrolled, of which consecutive 512 patients were included in the final analysis. Of these, 66 patients were excluded from the study for the following reasons: 30 patients did not have sufficient data for the study; 16 had iatrogenic post-procedural bleedings (endoscopic submucosal dissection for gastric tumors (*n* = 11), endoscopic mucosal resection (*n* = 2), endoscopic sphincterotomy (*n* = 3)), and 20 patients were lost to follow-up (Fig. 1).

The median age was 64 (range, 48–80 years) years old, and 71.9% patients were men. Among patients, 397 (69.7%) patients had comorbidities and 327 (36%) were taking anti-platelet agents or anti-coagulant medications on admission. The most common symptom of visiting the emergency center was melena (27.5%). The common causes of bleeding were gastric ulcer (32.8%), duodenal ulcer (20.5%), Mallory-Weiss tear (13.1%), and acute gastric mucosal lesion (12.9%) (Table 2).

**Table 2:** Baseline characteristics of patients with uppr gastrointestinal bleeding

Characteristics	*N* (%)
Overall	512 (100)
Median age (range), years	64 (48–80)
Male	368 (71.9)
Comorbidities;	
None	115 (30.3)
Hypertension	97 (18.9)
Diabetes mellitus	78 (15.2)
Cerebrovascular disease	56 (13.2)
Liver disease	47 (9.2)
Chronic renal impairment	40 (7.8)
Ischemic heart disease	30 (5.9)
Malignancy	26 (5.1)
Congestive cardiac failure	19 (3.7)
Chronic obstructive airways disease	17 (3.3)
Bleeding risk medications;	
None	327 (63.9)
Aspirin	101 (19.7)
Nonsteroidal anti-inflammatory drugs	38 (7.4)
Clopidogrel	35 (6.8)
Warfarin	11 (2.1)
Bleeding cause of endoscopic finding;	
Gastric ulcer	168 (32.8)
Duodenal ulcer	105 (20.5)
Mallory Weiss tear	67 (13.1)
Acute gastric mucosal lesion	66 (12.9)
Dieulafoy’s lesion	34 (6.6)
Gastrointestinal malignancy	34 (6.6)
Angiodysplasia	21 (4.1)
Esophageal ulcer	17 (3.3)
Serious clinical outcomes;	
Mortality	17 (3.3)
Rebleeding	65 (12.7)
ICU admission	86 (16.8)
Treatment;	
None	211 (41.2)
Argon plasma coagulation	216 (42.2)
Hemostatic forcep coagulation	30 (5.9)
Hemoclipping	55 (10.7)
Epinephrine injection	18 (3.5)
Embolization	7 (14)
Band ligation	1 (0.2)
Mean (95% CI) score;	
AIMS65 score	1.1 (0.1–2.1)
Pre-endoscopy Rockall score	3.1 (1.4–4.8)
Full Rockall score	5.6 (4.3–7.8)
Glasgow-Blatchford score	9.6 (5.4–13.6)

### Primary clinical outcome: *mortality*

Seventeen of the 512 patients (3.3%) died. Their median age was 70.24 (range 43–93) years old. The causes of death were uncontrolled bleeding (*n* = 5), complications due to cirrhosis (*n* = 5), sepsis due to pneumonia (*n* = 4), renal failure (*n* = 2), and cerebral infarction (*n* = 1). In the cases of 5 deaths due to uncontrolled bleeding, two patients underwent angiographic embolization and two patients underwent angiography followed by surgery. One patient died of active duodenal ulcer bleeding and hypovolemic shock during endoscopic therapy.

**Fig. 2:**
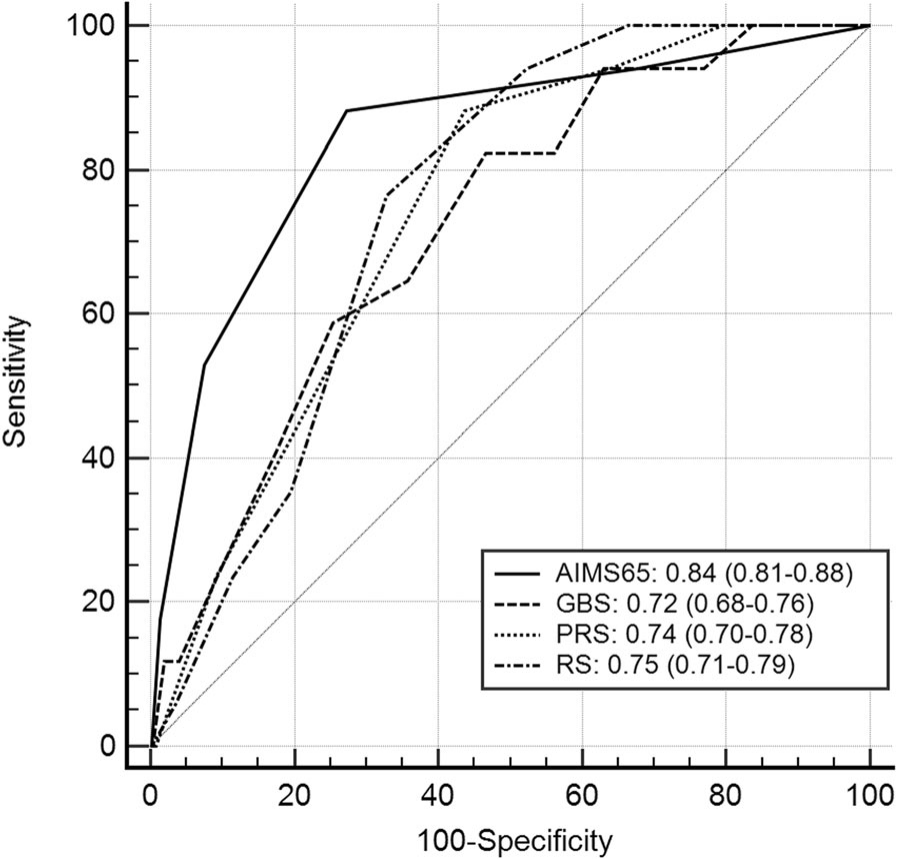
Area under the curve (AUC) of each scoring system for risk stratification scores as predictors of in-hospital mortality. The AIMS65 score (AUC 0.84, 95% CI, 0.81–0.88) seemed to be superior to the GBS (AUC 0.72, 95% CI, 0.68–0.76) for predicting mortality

All but one of the 17 patients who died had comorbidities. There was no difference between survivors and non-survivors in the use of anticoagulants. The mortality increased with increasing AIMS65 score, although death occurred in 1 patient who scored 0 on the AIMS65. Mortality was seen in 1/161 (0.6%) for AIMS65 0, 1/201 (0.5%) for AIMS65 1, 6/104 (5.8%) for AIMS65 2, 6/36 (16.7%) for AIMS65 3, 3/9 (33.3%) for AIMS65 4, 0/1 for AIMS65 5. The AUC values of each test were: AIMS65 = 0.84 (95% confidence interval (CI), 0.81–0.88), PRS = 0.74 (95% CI, 0.70–0.78), RS = 0.75 (95% CI, 0.71–0.79), and GBS = 0.72 (95% CI, 0.68–0.76). With regard to AUC value, there was a trend suggesting that the AIMS65 scoring system (0.84) seemed more accurate than the GBS system (0.72) for predicting mortality (*P* = 0.07) (Table 3) (Fig. 2).

**Table 3:** Comparison of AIMS65, GBS, Pre-endoscopic Rockall scores (PRS), and Rockall scores (RS) with significant clinical endpoints

	AUC (95% CI)	*P*-value of pairwise the AUC curves			
		AIMS65	PRS	RS	GBS
Mortality (*n* = 17)					
AIMS65	0.84 (0.81–0.88)	*	0.13	0.01	0.07
PRS	0.74 (0.70–0.78)	0.13	*	0.86	0.74
RS	0.75 (0.71–0.79)	0.09	0.86	*	0.65
GBS	0.72 (0.68–0.76)	0.07	0.74	0.66	*
Serious clinical outcomes (*n* = 134)					
AIMS65	0.68 (0.64–0.72)	*	0.54	0.52	0.40
PRS	0.66 (0.62–0.70)	0.53	*	0.05	0.76
RS	0.70 (0.65–0.74)	0.52	0.05	*	0.16
GBS	0.65 (0.61–0.70)	0.40	0.76	0.16	*
Rebleeding (*n* = 65)					
AIMS65	0.58 (0.54–0.62)	*	0.97	0.11	0.43
PRS	0.58 (0.54–0.62)	0.97	*	0.01	0.49
RS	0.63 (0.59–0.68)	0.11	0.01	*	0.04
GBS	0.55 (0.51–0.59)	0.43	0.49	0.04	*
ICU admission (*n* = 86)					
AIMS65	0.73 (0.69–0.77)	*	0.44	0.39	0.67
PRS	0.70 (0.66–0.74)	0.43	*	0.90	0.78
RS	0.70 (0.66–0.74)	0.39	0.90	*	0.72
GBS	0.71 (0.67–0.75)	0.67	0.78	0.72	*
Transfusion requirement (*n* = 397)					
AIMS65	0.69 (0.65–0.73)	*	0.95	0.06	< 0.001
PRS	0.70 (0.65–0.73)	0.95	*	0.01	< 0.001
RS	0.74 (0.70–0.77)	0.06	0.01	*	< 0.001
GBS	0.87 (0.84–0.90)	< 0.001	< 0.001	< 0.001	*

*PRS* Preendoscopic Rockall score, *RS* Rockall score

*Not available

### Secondary clinical outcomes

#### Composite serious clinical outcomes

Of the 512 patients, 134 (26.2%) were diagnosed with serious clinical outcomes (in-hospital mortality, rebleeding, or ICU admission). For these composite serious clinical outcomes, the AUC values of AIMS65, PRS, RS, and GBS were 0.68 (95% CI, 0.64–0.72), 0.70 (95% CI, 0.65–0.74), 0.66 (95% CI, 0.62–0.70), and 0.65 (95% CI, 0.61–0.70), respectively. AUC values of each scoring system did not differ significantly in terms of composite serious clinical outcomes.

#### Rebleeding

Rebleeding occurred in 65 patients (12.7%). Rebleeding occurred in patients with elderly, or chronic kidney disease. The AUC values for predicted rebleeding were as follows: AIMS65 = 0.58 (95% CI, 0.54–0.62), PRS = 0.58 (95% CI, 0.54–0.62), RS = 0.63 (95% CI, 0.59–0.67), and GBS = 0.55 (95% CI, 0.51–0.59). In pairwise comparisons between the scores for rebleeding, the AUC value of RS was superior to that of PRS and GBS (pairwise comparison, *P* = 0.01 and *P* = 0.04), but not statistically different than that of AIMS65 (pairwise comparison, *P* = 0.11).

#### ICU admission

Eighty-six patients (16.8%) were admitted to the ICU. The AUC values for predicted admission were: AIMS65 = 0.73 (95% CI, 0.69–0.77), PRS = 0.70 (95% CI, 0.66–0.74), RS = 0.70 (95% CI, 0.66–0.74), and GBS = 0.71 (95% CI, 0.67–0.75). All four scoring systems similarly predicted the need for ICU admission.

#### Transfusion requirements

Transfusion was required in 264 patients (62.3%) and the median transfusion was 2 units (interquartile range, 0–4). The AUC values for the need of transfusion were: AIMS65 = 0.69 (95% CI, 0.65–0.73), PRS = 0.70 (95% CI, 0.65–0.73), RS = 0.74 (95% CI, 0.70–0.77), and GBS = 0.87 (95% CI, 0.66–0.74). GBS was superior to other scoring systems in predicting transfusion requirement.

#### Endoscopic intervention

Endoscopic intervention was required in 301 patients (58.8%). The AUC values for the prediction of the need of endoscopic intervention were: AIMS65 = 0.57 (95% CI, 0.53–0.62), PRS = 0.56 (95% CI, 0.52–0.61), RS = 0.56 (95% CI, 0.52–0.61), and GBS = 0.61 (95% CI, 0.57–0.66) (Table 3).

### Cut-off value

The cut-off values for the endpoints of the risk stratification scores from the AIMS65, PRS, RS, and GBS were obtained when the cut-off value that maximized the sum of the sensitivity and the specificity was obtained. Sensitivity of AIMS65 was from 41.5% (CI 19.4–54.4, *p* < 0.001) to 88.2% (CI 63.6–98.5, *p* < 0.0001), which was similar to other scoring systems, ranging from 71.3 (CI 68.1–76.6, *p* < 0.001) to 78.6 (CI 74.1–82.6, *p* < 0.001), respectively. Cut-off as a value separating risk levels (high vs. low risk) for death was above 2 points on the AIMS65 and 8 points on the GBS, and 1 point on the AIMS65 and 11 points on the GBS for rebleeding. The cut-off values for ICU admission were 2 points on the AIMS65, 10 points on the GBS, 4 points on the PRS, and 5 points on the RS (Table 4).

**Table 4:** Cut-off values of each scoring system

Scoring system	Cut-off value	Sensitivity, % (95% CI)	Specificity, % (95% CI)	Low risk (%)	High risk (%)	*P*-value	Odds ratio (95% CI)
AIMS65							
Mortality	2	88.2 (63.6–98.5)	72.7 (68.6–76.6)	0.6	10	< 0.001	20.0 (4.5–88.6)
Serious clinical outcomes	2	51.5 (42.7–60.2)	78.6 (74.1–82.6)	18	46	< 0.001	3.8 (2.5–5.9)
Rebleeding	2	41.5 (29.4–54.4)	72.5 (68.1–76.6)	10.5	18	0.02	1.8 (1.1–3.2)
ICU admission	2	62.8 (51.7–73.0)	77.5 (73.2–81.3)	8.8	36	< 0.001	5.8 (3.5–9.5)
Transfusion requirement	1	35.5 (30.8–40.4)	92.2 (85.7–96.4)	62.1	84.6	< 0.001	3.3 (2.1–5.1)
Pre-endoscopic RS							
Mortality	4	88.2 (63.6–98.5)	56.4 (51.9–60.8)	0.6	4.8	0.01	3.3 (2.1–5.2)
Serious clinical outcomes	4	63.4 (54.7–71.6)	61.4 (56.3–66.3)	14.4	32.5	< 0.001	2.8 (1.7–4.5)
Rebleeding	3	76.9 (64.8–86.5)	36.9 (32.4–41.6)	8.3	15.1	0.03	1.9 (1.1–3.5)
ICU admission	4	72.1 (61.4–81.2)	60.3 (55.5–65.0)	7.2	22	< 0.001	3.6 (1.9–6.7)
Transfusion requirement	3	71.3 (66.6–75.7)	57.4 (47.8–66.6)	63.3	85.2	< 0.001	3.3 (2.1–5.1)
Rockall Score							
Mortality	7	76.5 (50.1–93.2)	67.1 (62.7–71.2)	1.2	7.4	< 0.001	6.6 (2.1–20.6)
Serious clinical outcomes	6	76.1 (68.0–83.1)	54.2 (49.1–59.3)	13.5	37.1	< 0.001	3.7 (2.4–5.9)
Rebleeding	6	72.3 (59.8–82.7)	48.9 (44.3–53.7)	7.6	17.1	< 0.001	2.5 (1.4–4.4)
ICU admission	7	60.5 (49.3–70.8)	70.9 (66.3–75.2)	10.1	29.5	< 0.001	3.7 (2.3–6.0)
Transfusion requirement	5	75.8 (71.3–80.0)	60.8 (51.3–69.8)	57.8	87	< 0.001	4.8 (3.1–7.5)
GBS							
Mortality	11	82.4 (56.6–96.2)	53.3 (48.8–57.8)	1.1	5.7	0.01	5.33 (1.5–18.7)
Serious clinical outcomes	10	75.4 (67.2–82.4)	49.5 (44.3–54.6)	15	34.6	< 0.001	3.00 (1.93–4.6)
Rebleeding	10	67.7 (54.9–78.8)	44.5 (39.8–49.3)	9.5	15.1	0.08^a^	1.6 (0.9–2.9)
ICU admission	11	75.6 (65.1–84.2)	57.8 (52.9–62.5)	7.9	26.5	< 0.001	4.2 (2.4–7.1)
Transfusion requirement	8	82.4 (78.3–86.0)	71.3 (62.1–79.4)	46.1	90.8	< 0.001	11.6 (7.1–18.7)

*P*-value denotes the Fisher exact test, which was used to compare low and high-risk groups

^a^Did not reach statistical significance

## Discussion

The AIMS65 scoring system was introduced to predict hospital mortality in patients with UGI bleeding based on clinical outcomes of bleeding has been validated for use in several studies [11, 15–18]. One study has found that the AIMS65 score is an independent predictor of mortality in patients with UGI bleeding [16]. One study showed that the accuracy of AIMS65 score is superior to that of GBS and PRS in predicting in-hospital mortality [16]. In contrast, one study showed that PRS is more useful for predicting mortality than the GBS and AIMS65 scores [18]. These results might be due to different patient characteristics and different mortality rate. The mortality rate was 3.3% in our study, compared to 4.2% in previous study [16], which suggests less severe patients were included in our study. It is important to compare the scoring systems within the same population and with a similar disease severity [12]. We conducted the present study in a large number of patients after excluding patients with variceal bleeding. The risk stratification score was calculated in all patients using the GBS, the PRS, and the RS. The AIMS65 showed similar performance to the GBS and the RS in predicting mortality, rebleeding, ICU admission, and endoscopic intervention.

The risk stratification scores were recommended for management of prognosis and serious outcomes for NVUGI bleeding [19]. In order to be an effective tool for risk stratification, a measure should be easy to use and accurately predict bleeding outcomes [16]. For example, the CHADS2 scoring systems, which is used to predict cerebral vascular risk in patients with atrial fibrillation, is widely used because of its accessibility and simplicity [20]. The GBS scoring system is limited by weighting, which makes calculation difficult. RS scoring system also has limitations. Weighting leads to complexity in calculation and it requires endoscopic data for calculation, impossible to apply at the time of presentation. Those systems are much more difficult to apply by the busy clinician in routine clinical practice due to their complexity [21]. AIMS65 has only 5 components (albumin, INR, mental status, systolic BP, age) and each component is the same value of 1 point. The recently-developed AIMS65 is much easier to apply in clinical practice.

In one study, GBS score 0 identified low-risk patients who can be managed safely as outpatients [22]. Another study showed lower-risk patients (GBS score < 12) who were taken urgently to endoscopy were related to the worse outcomes [23]. In our study, patients with AIMS65 score 0/1 had low risk of mortality of 0.5%. We believe that AIMS65 score 0/1 provides a rationale for delaying emergency endoscopic intervention until the next day when patients arrive in the evening or night time.

Several studies validated the performance of AIMS65 scoring system to predict the clinical outcomes in patients with UGI bleeding in Korea. One study showed that AIMS65 was not useful for predicting the need for endoscopic intervention and transfusion in Korea [14]. In this study, 22% (64/286) patients with variceal bleeding were included. AIMS65 score showed lower performance than GBS and RS regardless of variceal or non-variceal bleeding group. On the other hand, the other study involving 523 patients with NVUIB showed that AIMS65 score was useful for predicting the mortality, transfusion requirement, and endoscopic intervention in Korean patients with NVUIB [11]. This difference might be due to different patient characteristics and patient number.

There are limitations in this study. The study design was retrospective. However, the data were independently reviewed by two co-authors. We tried to minimize errors by collecting all medical records for NVUGI bleeding patients.

Despite the large number of patients included in this study, mortality occurred in only 17 patients (3.3%). Each scoring system to predict mortality was less accurate than previously reported, because death events were rare [7]. Another limitation of the study is single center study.

## Conclusion

The AIMS65 score in NVUGI bleeding patients was comparable to the GBS or Rockall scoring systems when predicting mortality, rebleeding, ICU admission, and endoscopic intervention in Korean patients. The AIMS65 score, however, is much easier to calculate using variables routinely available in the emergency clinical situation, and has the advantage that it can be performed by the busy clinician before an endoscopy. Therefore, we recommend AIMS65 for prediction of severity of GI bleeding in daily practice. Before the AIMS65 becomes a standard of care for the risk stratification of UGI bleeding cases, further multicenter prospective studies will be required.

## Data Availability

Data for the analyses are available from the corresponding author on request.

## Abbreviations

AUC: Area under the curve
GBS: Glasgow-Blatchford score
ICU: Intensive care unit
NVUGI: Non-variceal upper gastrointestinal
PRS: Pre-endoscopy Rockall score
ROC: Receiver-operating curve
RS: Rockall score
